# Co-Occurring Genetic Mutations in Rett Syndrome and *MECP2*-Related Disorders—Clinical and Diagnostic Implications from a Case Series

**DOI:** 10.3390/genes17030274

**Published:** 2026-02-27

**Authors:** Jatinder Singh, Samiya Chishti, Paramala Santosh

**Affiliations:** 1 Department of Child and Adolescent Psychiatry, Institute of Psychiatry, Psychology and Neuroscience, King’s College London, London SE5 8AF, UK; 2 Centre for Interventional Paediatric Psychopharmacology and Rare Diseases (CIPPRD), South London and Maudsley NHS Foundation Trust, London SE5 8AZ, UK

**Keywords:** case series, multiple genetic diagnoses, Rett syndrome, *MECP2*-related disorders, neurodevelopmental phenotypes

## Abstract

**Background/Objectives**: Factors modulating phenotypic variability in Rett syndrome (RTT, OMIM 312750) include X chromosome inactivation (XCI), type of *MECP2* variant, and/or disease modifiers. Emerging evidence also points to multi-locus genetic variants. Understanding the phenotypic variability associated with multi-locus genetic diagnoses in individuals with RTT and *MECP2*-related disorders would be important not only for accurate diagnosis, risk stratification and clinical management but also to explain symptoms that might not be typically associated with RTT. **Methods**: We present a case series of five individuals with a diagnosis of RTT or an *MECP2*-related disorder with co-occurring genetic findings, including pathogenic variants, variants of unknown significance and chromosome duplications. Clinical features such as neurodevelopmental history and comorbid medical conditions were assessed alongside the genetic findings. **Results**: A review of 200 cases with RTT identified five cases (all females aged 7–27 years) with a co-occurring genetic finding. Each case harboured at least one additional genetic variant that included a beta thalassaemia trait, *Calmodulin 3* (*CALM3*) missense variant, maternally inherited 22q12.3 to q13.1 duplication, 7p14.3 and *Dynein Cytoplasmic 1 Heavy Chain 1* (*DYNC1H1*) variants of uncertain significance and a pathogenic *Set Domain-containing protein 5* (*SETD5*) variant. A rare triple genetic finding was illustrated in a single case, combining *MECP2*, *CALM3*, and *DYNC1H1* variants. **Conclusions:** This case series supports the premise that RTT and *MECP2*-related disorders exist in a more complex neurogenetic spectrum than previously defined. It also emphasises the complexity within *MECP2*-related disorders. They are not static, and in the context of severe treatment resistant epilepsy, *MECP2* disorders can evolve over time, necessitating diagnostic reclassification. Although the co-occurrence of multiple genetic disorders in RTT and *MECP2*-related disorders is rare, these cases underscore the importance of considering cumulative genetic burden when evaluating individuals with atypical features or evolving neurodevelopmental phenotypes.

## 1. Introduction

Rett syndrome (RTT) (OMIM #312750) is an X-linked neurodevelopmental disorder that, in most cases (~90%), is due to a pathogenic variant in the gene *methyl-CpG binding protein 2* (*MECP2*), leading to a defective protein. It remains a clinical diagnosis [1]. There is a wide phenotypic spectrum in RTT, and some individuals may have a pathogenic *MECP2* variant but show no signs of the disorder [2]. The genetics of RTT are not necessarily predictive of the phenotype [3,4,5]. However, evidence from cross-sectional and longitudinal studies [6,7] suggest associations between genotype and clinical phenotype in RTT, indicating that genotype is likely an influencing factor. As in some other X-linked disorders, such as Fabry disease [8], X-chromosome inactivation in RTT cannot fully account for the clinical heterogeneity. We and others have previously surmised that multi-loci genetic contributions may play an important role [4,9,10,11,12]. In a cohort of 65 individuals with RTT, we showed that a subset of individuals (26%) had homozygotic *5,10-methylenetetrahydrofolate reductase* (*MTHFR*) polymorphisms rs1801133 and rs1801131 associated with reduced MTHFR activity [9]. The rs1801133 and rs1801131 polymorphisms can influence disease phenotype in RTT; however, these *MTHFR* polymorphisms are common population variants and are not typically classified as pathogenic variants with high penetrance causing monogenic disease. Extending the phenotype of a single gene disorder to a novel syndrome should be viewed with caution, as highlighted in the case of Fitzsimmons syndrome [13], and must be more broadly considered in the context of monogenic neurodevelopmental disorders that may harbour pathogenic or likely pathogenic variants in other genes.

In individuals with RTT, it is prudent to test for other genetic variants when a pathogenic *MECP2* variant has not been identified [14,15]. However, what is not widely known is the impact of an additional genetic diagnosis in RTT. Recent information suggests that the diagnostic yield ranges from 19% to 56% in rare diseases [16], and the prevalence of multiple molecular diagnoses in an individual is 0.7–7.5%, with a recent estimate at 9% [16]. In complex disorders such as RTT, whole-genome sequencing (WGS) may miss an additional genetic diagnosis. Adopting a specialised, multidisciplinary approach may help optimise rare disease diagnosis from WGS [17]. Multiple genetic findings are also suggested to be more common in individuals with more clinical complexity [18] and exist in the sphere of developmental disorders [19]. Ridsdale et al. (2024) showed that dual diagnoses of genes associated with synergistic phenotypes manifest as more severe clinical phenotypes than those involving antagonistic phenotypes [19]. The synergistic phenotypes were more associated with intellectual disability and developmental delay. Dual genetic rare diseases have also been documented in five paediatric patients, resulting in 15 genetic variants and two copy number variants (CNVs) [20], further strengthening the need to consider additional genetic variants when assessing complex neurodevelopmental phenotypes.

Dual genetic diagnosis has been coined as ‘double trouble’ [21]. The previous literature has identified two studies of individuals with ‘double trouble’ or multi-loci genetic contributions including pathogenic *MECP2* variants and 15q11-13 rearrangements [22] or *MECP2* with Ephrin receptor B4 (*EPHB4*) in an 8-year-old girl presenting with RTT-related symptoms and coexisting vascular abnormalities [23]. These results indicate that dual genetic diagnoses, although rare, can contribute to phenotypic variability in RTT and related disorders. While the concept of multiple genetic diagnoses in an individual is challenging [20,24], the few documented cases described in the literature underscore the notion that there is a significant knowledge gap in our understanding of multiple genetic diagnoses in RTT and the potential impact this may have on clinical phenotype. Given the scarcity in the existing literature, a case series affords a timely opportunity to describe the clinical features of individuals with RTT and *MECP2*-related disorders who harbour an additional genetic diagnosis. The main aims of this case series are therefore threefold: (I) to describe the phenotypic variability associated with a co-occurring genetic diagnosis in individuals with RTT and a *MECP2*-related disorder, (II) to improve our knowledge of their potential contribution to phenotypic complexity, and (III) to assess patterns in atypical features.

## 2. Methods

### 2.1. Participants and Data Collection

In 2025, the electronic health records of 200 individuals with RTT that had been previously referred to the Centre for Interventional Paediatric Psychopharmacology and Rare Diseases were retrospectively reviewed. Excluding those individuals with *MTHFR* and *BDNF* polymorphisms as previously described [4,9], we identified five cases that met (I) a diagnosis of RTT (clinical and/or genetic) and (II) had an additional genetic diagnosis. This information was identified from reports from Clinical Genetic Centres or from other electronic health records. The data collection also included the age, gender, and clinical manifestations.

### 2.2. Variant Detection

Genetic testing was undertaken using a variety of approaches. For Case 2, a pathogenic *MECP2* variant was identified by the PanelApp genetic epilepsy syndromes v2.2 gene panel using next-generation sequencing (Twist Core Human Exome/Illumina NextSeq). For Case 4, the 7p14.3 gain of uncertain significance was determined using single-nucleotide polymorphisms (SNP) array analysis. In Case 5, *MECP2* testing was performed using the intellectual disability (R29.4) GMS panel v3.2 using whole-genome sequencing (Illumina). Information on the genetic testing methods was not available for Cases 1 and 3.

### 2.3. Data Management and Analysis

For each case, data were extracted into a table by the first author (J.S.) and then independently verified with the electronic health records by the second author (S.C.). A description of the cases was performed by both J.S. and S.C. and reviewed by the third author, P.S. Demographic data for each case were tabulated.

### 2.4. Informed Consent and Ethics Approval

This study forms part of the Tailored Rett Intervention and Assessment Longitudinal (TRIAL) database, which obtained ethics approval on 12 December 2023 (REC reference: 15/LO/1772). Caregivers gave verbal consent to participate in the study for research and publications as stated in the 15/LO/1772 protocol.

## 3. Results

We reviewed 200 RTT cases and identified five individuals with a co-occurring genetic finding. The case series included five individuals aged 7 to 27 years with RTT or a *MECP2*-related disorder (*MECP2* epileptic encephalopathy—see description of Case 5). All cases had a confirmed diagnosis of RTT. Three had frameshift mutations of the *MECP2* gene (Cases 1, 2 and 5) while another (Case 3) had a nonsense mutation. For Case 4, the RTT diagnosis was atypical. Alongside these *de novo MECP2* pathogenic variants, each individual harboured at least one additional genetic variant, and in Case 2, a triple genetic diagnosis was identified. The genetic variants included a beta thalassaemia trait, a *Calmodulin 3* (*CALM3*) missense variant, a maternally inherited 22q12.3 to q13.1 duplication, 7p14.3 and *Dynein Cytoplasmic 1 Heavy Chain 1* (*DYNC1H1*) variants of uncertain significance, and a pathogenic *Set Domain-containing protein 5* (*SETD5*) variant. These observations show that dual and multiple genetic diagnoses in individuals with RTT comprise pathogenic variants, variants of unknown significance (VUS) and a chromosome duplication (Table 1).

### Characteristics of the Case Series

*Case 1: RTT and a beta thalassaemia trait.*

Case 1 was a 27-year-old female who was referred to the CIPPRD in June 2023 for psychopharmacological intervention in relation to symptoms of anxiety and low mood in the context of RTT. The individual had longstanding symptoms of RTT alongside multiple joint pain, deterioration in her ability to walk independently and generalised anxiety disorder. From the medical notes, there was no evidence of autoimmune connective tissue disease, and she had received a diagnosis of fibromyalgia/chronic pain syndrome and polyarthropathy. The individual was diagnosed with osteoporosis after undergoing a DEXA scan in 2023. Swallowing problems were also noted. The electronic health records described a beta thalassaemia trait and associated mild anaemia; however, further details regarding the clinical impact of the beta thalassaemia trait, including other haematological parameters, were not available. Beta thalassaemias are characterised as a group of hereditary blood disorders with reduced or absent synthesis in the beta globin chain, causing decreased haemoglobin in red blood cells [25]. While the importance of a beta thalassaemia trait in the context of anaemia in pregnancy is well-established, some data have suggested an increase in mild symptoms of anaemia and episodes of pyrexia in individuals with a beta thalassaemia trait [26]. However, given the high prevalence of monogenic haemoglobin disorders in the broader population, the generally mild presentation of beta thalassaemia traits [26], and the absence of previously reported associations between a beta thalassaemia trait and neurodevelopmental disorders, the clinical significance of a beta thalassaemia trait remains unknown. Therefore, the co-occurrence with RTT in this case is unclear, and no clinical inferences can be made. Nevertheless, to the best of our knowledge, this is the first documented case of a beta thalassaemia trait associated with RTT.

*Case 2: RTT, CALM3 and DYNC1H1 variants.*

In February 2023, a 7-year-old child was referred to the CIPPRD following concerns about symptoms of behavioural dysregulation. The young girl also had epilepsy, dystonia, gastroesophageal reflux disease and constipation. At 18 months, the child showed signs of neurological regression and was initially clinically diagnosed with RTT, and genetic testing supplemented this diagnosis. The child was referred for genetic testing in 2022 to investigate a clinical presentation with branchial fistula, squint with reduced vision, and developmental delay. Genetic testing confirmed a heterozygous pathogenic *MECP2* variant (c.1164_1207del p.(Pro389Ter). Further genetic testing revealed that the child was also heterozygous for the *Dynein Cytoplasmic 1 Heavy Chain 1* (*DYNC1H1*) variant of uncertain significance (VUS). More recent genetic testing in 2025 identified a new genetic diagnosis of Long QT syndrome due to a pathogenic *calmodulin 3* (*CALM3*) mutation (missense variant–p[Asp132His]). These findings illustrate a complex case presentation against the backdrop of a pathogenic *MECP2* variant with co-occurrence of *DYNC1H1* VUS and a pathogenic *CALM3* variant associated with Long QT syndrome. The latter would be particularly important because RTT is already associated with an increased risk of QT prolongation [27,28,29], and the co-occurrence of a pathogenic *CALM3* variant may further potentiate this risk. Pathogenic variants of *CALM3* are associated with extreme QT prolongation (QTc > 650 ms) [30,31], and some *CALM* variants can cause overlapping cardiac anomalies such as catecholaminergic polymorphic ventricular tachycardia (CPVT) and Long QT [32]. This case highlights the intersection between pathogenic *MECP2* and *CALM3* variants and the heightened arrhythmic risk they confer. It reinforces the importance of ongoing cardiac monitoring in this young girl. While the heterozygous *DYNC1H1* variant in this child lacks sufficient evidence to be classified as pathogenic, evidence from 47 cases with pathogenic heterozygous variants in *DYNC1H1* points to multisystem impairments, including cortical malformations, intellectual disability, immunodeficiency, skeletal problems, and hearing loss [33]. Observations from case studies further suggest that heterozygous pathogenic variants in *DYNC1H1* are also implicated in neuromuscular disorders [34]. When viewed together, the coexistence of *MECP2*, *CALM3* and a *DYNC1H1* VUS in this girl highlights a unique triple genetic presentation with implications for both cardiac and neurodevelopmental risk.

*Case 3: RTT and maternally inherited 22q12.3 to q13 duplication.*

This 12-year-old girl, known to have a history of uncontrolled epilepsy, dystonia, breathing difficulties, scoliosis and constipation, was referred to the CIPPRD in May 2024 due to behavioural dysregulation. There was also a history of pancreatitis, pleural effusion and hiatal hernia (surgically repaired). She was previously referred for *MECP2* gene analysis due to motor development delay and small stature. Sequence analysis of exon 4 of the *MECP2* gene revealed a heterozygous C to T base substitution at nucleotide position 763 (c.763C > T) causing a substitution in arginine at position 255 for a stop codon p.(Arg255*). Additional genetic testing detected a maternal duplication of approximately 883kb containing 33 genes at chromosome 22 between bands q12.3 and q13.1. This region is not recorded as a benign copy number variant (CNV) in the Database of Genomic Variants (DGV). Duplications in this region can cause short stature and intellectual disability; however, the clinical phenotype is highly variable [35]. The information in the case suggests that although the maternally inherited 22q12.3 to q13 duplication can be associated with neurodevelopmental abnormalities in offspring, the lack of the clinical phenotype in the mother suggests reduced penetrance or variable expression. Hence, the clinical significance of a co-occurring duplication of approximately 883kb on chromosome 22 in this child is unclear, highlighting the challenges of interpreting multiple genetic factors in clinical practice.

*Case 4: RTT and 7p14.3 gain*.

This case was an 8-year-old girl whose concerns were first noted at 1 year of age. Previously, there was no loss of skills, and the girl was reported to have made gradual progress sitting unsupported until 2 years of age. She was unable to crawl or weight-bear but was able to roll from her side; however, she did not gain the ability to walk. Speech and language development was also reported as severely delayed, and she did not gain the ability to talk. A diagnosis of RTT was made, but it did not meet the diagnostic criteria for classical RTT. The electrographic findings showed slow background and epileptiform discharges. She was referred to the CIPPRD in February 2023 due to concerns regarding symptoms of anxiety and sleep disturbance.

The clinical letter identified a 46kb interstitial heterozygous copy number gain on the short arm of chromosome 7 in the region 14.3 (7p14.3 [33133742_33179557] x3). This region is comorbid with two OMIM genes, *RP9 pre-mRNA splicing factor* (*RP9*, OMIM: 607331) [36] and *PTH-responsive B1 gene* (*BBS9*, OMIM: 607968) [37], with the corresponding gene phenotypes: Retinitis pigmentosa 9 and Bardet-Biedl syndrome 9. The suggested gene–phenotype relationship for Retinitis pigmentosa 9 is provisional. Bardet–Biedl syndrome 9 (BBS9) is a rare multisystem disorder characterised by intellectual disability, obesity, renal abnormalities, facial dysmorphism, and short stature [37]. Given (I) the absence of documented evidence supporting genotype–phenotype correlations due to changes in the *RP9* gene and (II) that the inheritance pattern for pathogenic *BBS9* variants is mainly autosomal recessive [38,39], the copy number gain at 7p14.3 was classified as a variant of uncertain clinical significance. At present, there is insufficient information to demonstrate this genetic change as disease-causing. In summary, this case shows that although the clinical presentation was not entirely consistent with classical RTT, the copy number gain at 7p14.3 is unlikely to contribute to the RTT-like phenotype.

*Case 5: MECP2 Epileptic Encephalopathy and SETD5*

Case 5 was a 15-year-old female with pathogenic variants in the *MECP2* and *SETD5* genes. She had global developmental delay and complex epilepsy associated with four different seizure types, with the most common being focal epilepsy, and was able to walk at two years of age but had learning difficulties. A brain MRI showed diffuse sub-cortical atrophy mainly in the frontal lobe and brain stem. She was referred to the CIPPRD in September 2023 due to concerns regarding focal and tonic–clonic seizures, gait instability, jerky movements of the hands and arms, reduced verbal communication with poor engagement and double incontinence. Genetic testing to investigate the cause of the clinical presentation identified a heterozygous *de novo* pathogenic *MECP2* variant (c.997_1013del;1148_1168del). In October 2022, she received a diagnosis of atypical RTT. However, following periods of regression associated with poorly controlled epilepsy, the young female lost her ability to walk and talk. In July 2025, her diagnosis was revised to *MECP2*-related epileptic encephalopathy. This case highlights the diagnostic complexity within *MECP2*-related disorders. It is not static and shows that in the context of severe treatment-resistant epilepsy, *MECP2* disorders can evolve over time and necessitate diagnostic reclassification. This young female also had a *de novo* pathogenic *SETD5* variant (c.941A > G). This gene encodes a protein lysine-methyltransferase that plays a key role in regulating gene expression [40]. Defects in the *SETD5* gene disrupt epigenetic mechanisms. Monoallelic pathogenic *SETD5* variants cause intellectual developmental disorder (OMIM: 615761) [41], and variants have also been reported in other genetic neurodevelopmental disorders such as KBG syndrome and Cornelia de Lange syndrome [40]. The co-occurrence of a de novo *SETD5* pathogenic variant could suggest a disease-modifier effect contributing to early developmental vulnerability and epilepsy complexity. The dual diagnosis reaffirms the need to carefully consider genetic interactions, particularly when clinical progression deviates from expected diagnostic trajectories, especially in atypical neurodevelopmental presentations.

## 4. Discussion

The five cases presented illustrate the clinical and genetic variability in RTT and *MECP2*-related disorders. It shows that additional genetic variants, CNV and comorbid medical conditions can influence phenotype, disease outcomes and clinical management. The presence of co-occurring pathogenic variants, VUS and chromosomal duplications also highlights the limitations of a single-gene diagnostic framework in RTT. The case series further emphasise that *MECP2*-related disorders are not static and can evolve over time, particularly in the context of refractory epilepsy. An accumulator effect may also be relevant for synergistic genes involved in epigenetic regulation (*SETD5*), cytoskeletal transport (*DYNC1H1)*, and cardiac ion signalling (*CALM3*), which overlap with neurodevelopment pathways and could confer additional neurodevelopmental vulnerability. Synergistic genes are more likely to be associated with intellectual disability or developmental delay phenotypes [19]. The intersection of pathogenic *MECP2* variants and genes involved in cardiac risk also reinforces the notion for proactive cardiac surveillance in RTT. When viewed together, this raises the importance of comprehensive genomic evaluation and longitudinal clinical assessment in RTT to support accurate diagnosis and risk stratification, especially in individuals with unusual or complex presentations. This case series highlights those variants with plausible biological interactions with *MECP2*. For instance, in Case 2, the co-occurring *CALM3* variant may interact with the MECP2-driven risk for QT prolongation, potentially amplifying cardiac risk. Moreover, in Case 5, the *SETD5* variant may exacerbate neurodevelopmental vulnerability by overlapping with *MECP2* gene-regulatory pathways. However, these cases should also be viewed through the lens of coincidental findings where the penetrance or phenotypic impact is uncertain, as observed with the maternally inherited 22q12.3 to q13 duplication (Case 3) and the 7p14.3 gain (Case 4). It is important to consider information from other spheres that suggest a cautious approach when interpreting primary versus secondary genetic findings in monogenic diseases [42].

Hypoxia and/or immune-based factors may also modify the genetic outcomes of disease. In the context of hypoxia, an underlying genetic defect itself [43] may predispose individuals to sustaining a hypoxic–ischaemic injury at birth, which can worsen neurodevelopmental outcomes. Evidence has also shown that in infants with hypoxic–ischaemic encephalopathy (HEI) and an additional genetic anomaly, there was more neurodevelopmental impairment and a higher rate of mortality when compared to infants with HEI [44]. These findings are important given that in a child with HEI, the presence of a genetic diagnosis may not be apparent at birth, supporting a lower threshold of genetic testing in a child with HEI [44]. Beyond hypoxia, immune-based factors [45] can also play a role in worsening outcomes in individuals with underlying genetic vulnerability. Neuropsychiatric and immune-based disorders share common molecular pathways, and genetic risk for psychiatric disorders may also be linked to genetic risk of immune disorders [46]. Emerging evidence in 22q11.2 deletion syndrome (DiGeorge Syndrome) suggests that neuroinflammatory processes may interact with genetic risk to influence neurodevelopmental trajectory [47], and a cross-trait genome-wide association study (GWAS) meta-analysis has revealed that a subset of individuals may have a higher risk for neurodevelopmental and immune-based disorders [48]. Recent case reports have illustrated a dual diagnosis at the neuroimmune axis [49,50]; however, whether comparable neuroimmune interactions extend to RTT remains to be established and requires further investigation. When viewed collectively, these factors suggest that an integrated model of care which considers genetic vulnerability alongside modifying factors could be more beneficial for informing diagnosis and clinical management.

## 5. Limitations

Our case series was small with only five cases, and therefore, the observed patterns may not reflect the wider RTT population. For Cases 1 and 4, there was incomplete clinical data, which limits our ability to provide a more robust clinical picture across the cases. Two of the cases (Case 2 and Case 4) also had VUS, therefore making it difficult to attribute clinical features to a known pathogenic profile. We are also mindful that treatment-related epigenetic influences and/or immune-based factors may also contribute to disease progression. These potential confounding factors were not captured in our case series and could have a significant impact on clinical variability. There was also limited information on the genetic testing approaches used. Despite these limitations in the current case series, recognition of multi-locus contributions in RTT and *MECP2*-related disorders underscores the need for genomic analysis and multidisciplinary management. Integration of additional genetic findings could help to optimise personalise care in this vulnerable patient population.

## 6. Conclusions

The diversity of genetic findings in this case series supports the growing recognition that RTT and *MECP2*-related disorders exist in a more complex neurogenetic ecosystem than previously defined and may complicate clinical interpretation. Although primarily descriptive, some mechanistic details can be gleaned from this case series. The mutation in calmodulin 3, a key protein involved in cardiac ion channel regulation, could provide a mechanistic basis for the increased risk of QT prolongation observed in Case 2. Furthermore, variants in *SETD5* described in Case 3 suggest biological convergence with another gene, *MECP2*, involved in gene regulation, potentially amplifying neurodevelopmental severity. While the co-occurrence of multiple genetic disorders in RTT and *MECP2*-related disorders is rare, it would be important to consider the cumulative genetic burden in cases with atypical features or evolving neurological phenotypes. In conclusion, this case series shows that a single genetic disorder should not be viewed as the sole explanatory factor in complex neurodevelopmental disorders and that broader genomic evaluation may be informative.

## Figures and Tables

**Table 1:** Description of five cases with additional genetic diagnosis.

Clinical Information	Case 1	Case 2	Case 3	Case 4	Case 5
Age * (years)	27	7	12	8	15
**Age (years) at which *MECP2* mutation identified**	24	3	~2	1	11
**Gender**	F	F	F	F	F
**Deviations from core Rett syndrome phenotype**	Fibromyalgia/chronic pain syndrome Haematological comorbidity	Branchial fistula Malignant QTc	Pancreatitis Pleural effusion Hiatal hernia	No defined period of developmental regression	No defined period of developmental regression in the initial stage Regression noted at the age of 14 years
**Clinical characteristics that are potentially genetic modifier driven**	Unusual fatigue and tiredness Polyarthropathy	Extreme QT prolongation	Uncertain	Uncertain	Uncertain
**Rett syndrome**	*MECP2* heterozygous pathogenic variant NM_004992.3:c.1164_1207del p.(Pro389*)n	*MECP2* pathogenic variant NM_004992.3:c.1164_1207del p.(Pro389Ter)	*MECP2* heterozygous pathogenic nonsense mutation c.763C > T; p.(Arg255*) detected in exon 4	Yes (atypical RTT)	*MECP2* heterozygous pathogenic variant NM_004992.3:c.997_1013del;1148_1168del p.[Gly333Leufs*2;Leu383_Pro389del]
**Additional genetic mutation**	Beta thalassaemia trait	Pathogenic *CALM3* variant (missense variant–p[Asp132His]). Heterozygous *DYNC1H1* variant NM_001376.4:c.5433_5433 + 27dup-variant of uncertain significance	Maternally inherited duplication at 22q12.3 to q13.1	7p14.3 gain of uncertain significance (7p14.3 [33133742_33179557] x3)	*SETD5* heterozygous pathogenic variant NM_001080517.3:c.941A > G p.(Asn314Ser)

* Age of individual and not when diagnosis was made. Abbreviations: *CALM3* (*gene encoding Calmodulin 3 protein*); *DYNC1H1* (*gene encoding Dynein Cytoplasmic 1 Heavy Chain 1 protein*); *MECP2* (*gene encoding Methyl-CpG Binding Protein 2*); *SETD5* (*Set Domain-containing protein 5*).

## Data Availability

The original contributions presented in this study are included in the article. Further inquiries can be directed to the corresponding author.
